# BTK抑制剂联合R-CHOP方案治疗初诊双表达弥漫大B细胞淋巴瘤的疗效与安全性观察

**DOI:** 10.3760/cma.j.cn121090-20250811-00375

**Published:** 2026-02

**Authors:** 锦波 卢, 蕾 曹, 海玲 刘, 祎 缪, 浩睿 申, 奕 夏, 华 尹, 月新 程, 建勇 李, 磊 范

**Affiliations:** 1 南京医科大学第一附属医院（江苏省人民医院）血液科，南京 210029 Department of Hematology, the First Affiliated Hospital of Nanjing Medical University（Jiangsu Province Hospital）, Nanjing 210029, China; 2 盐城市第一人民医院血液科，盐城 224000 Department of Hematology, Yancheng First People's Hospital, Yancheng 224000, China

**Keywords:** 淋巴瘤，大B细胞，弥漫性, 布鲁顿酪氨酸激酶抑制剂, R-CHOP, 疗效, 安全性, Lymphoma, large B-cell, diffuse, Bruton tyrosine kinase inhibitor, R-CHOP, Efficacy, Safety

## Abstract

**目的:**

探讨布鲁顿酪氨酸激酶抑制剂（BTKi）联合R-CHOP方案治疗初诊双表达（DE）弥漫大B细胞淋巴瘤（DLBCL）的疗效与安全性。

**方法:**

回顾性分析2021年6月至2024年6月期间南京医科大学第一附属医院收治的95例确诊为DLBCL且免疫组织化学染色判断MYC蛋白表达≥40％，BCL2蛋白表达≥50％的DE患者临床资料。其中35例接受BTKi（泽布替尼/奥布替尼/阿可替尼）联合R-CHOP方案治疗（BTKi+R-CHOP组），60例接受R-CHOP方案治疗（R-CHOP组）。观测指标包括客观缓解率（ORR）、完全缓解（CR）率、无进展生存（PFS）、总生存（OS）以及安全性评估。

**结果:**

BTKi+R-CHOP组患者中位年龄61（29～73）岁。诱导治疗结束时，BTKi+R-CHOP组ORR和CR率均高于R-CHOP组（ORR：94.3％对71.7％，*P*＝0.008；CR率：91.4％对65.0％，*P*＝0.006）。中位随访30（6～47）个月，BTKi+R-CHOP组1年及2年PFS率分别为88.5％（95％ *CI*：78.5％～99.8％）和85.1％（95％ *CI*：73.8％～98.1％），1年及2年OS率均为94.3％（95％ *CI*：86.9％～100％）。R-CHOP组1年、2年PFS率分别为61.7％（95％ *CI*：50.5％～75.3％）和48.8％（95％*CI*：37.4％～63.6％）；1年、2年OS率分别为80.0％（95％ *CI*：70.5％～90.8％）和65.1％（95％ *CI*：53.7％～78.8％）。两组生存差异均具有统计学意义（PFS：*P*＝0.002；OS：*P*＝0.010）。亚组分析显示，BTKi+R-CHOP组在不同临床特征患者中均获得较高缓解率，各亚组ORR均>85％。BTKi联合R-CHOP方案治疗期间≥3级不良事件主要为中性粒细胞减少症（28.6％）和肺部感染（14.3％），无致死性出血或心血管事件。

**结论:**

BTKi联合R-CHOP方案可显著提高DE-DLBCL患者的缓解率并增加生存获益，安全性可控。

## Abstract (en)

**Objective:**

To evaluate the efficacy and safety of Bruton tyrosine kinase inhibitor（BTKi）combined with R-CHOP regimen in newly diagnosed double-expressor diffuse large B-cell lymphoma（DE-DLBCL）.

**Methods:**

A retrospective analysis was conducted on 95 patients with DLBCL meeting DE criteria by immunohistochemistry（MYC protein expression ≥ 40％, BCL-2 protein expression ≥ 50％）treated at the First Affiliated Hospital of Nanjing Medical University between June 2021 and June 2024. Among them, 35 patients received BTKi（zanubrutinib, orelabrutinib, acalabrutinib）combined with R-CHOP（BTKi + R-CHOP group）, and 60 received R-CHOP regimen alone（R-CHOP group）. Outcomes included objective response rate（ORR）, complete response（CR）rate, progression-free survival（PFS）, overall survival（OS）, and safety.

**Results:**

The median age of the BTKi+R-CHOP group was 61（range, 29–73）years. At the end of induction therapy, the BTKi+R-CHOP group had a higher ORR（94.3％ *vs* 71.7％, *P*＝0.008）and CR rate（91.4％ *vs* 65.0％, *P*＝0.006）than the R-CHOP group. After a median follow-up of 30（range, 6–47）months, the 1- and 2-year PFS rates in the BTKi+R-CHOP group were 88.5％（95％ *CI*: 78.5％–99.8％）and 85.1％（95％ *CI*: 73.8％–98.1％）, respectively, and the 1- and 2-year OS rate was 94.3％（95％ *CI*: 86.9％–100％）. In the R-CHOP group, the 1-year and 2-year progression-free survival（PFS）rates were 61.7％（95％ *CI*: 50.5％–75.3％）and 48.8％（95％ *CI*: 37.4％–63.6％）, respectively; the 1-year and 2-year overall survival（OS）rates were 80.0％（95％ *CI*: 70.5％–90.8％）and 65.1％（95％ *CI*: 53.7％–78.8％）, respectively. Between-group differences were significant for both PFS（*P*＝0.002）and OS（*P*＝0.010）. Subgroup analyses showed high ORRs across clinical subgroups in the BTKi+R-CHOP group（all ORR>85％）. Grade ≥ 3 adverse events primarily included neutropenia（28.6％）and pulmonary infection（14.3％）; no fatal bleeding or cardiovascular events occurred.

**Conclusion:**

BTKi combined with R-CHOP significantly improved response rates and survival in patients with DE-DLBCL, with manageable safety.

弥漫大B细胞淋巴瘤（DLBCL）是一组高度异质性的侵袭性淋巴瘤，占成人非霍奇金淋巴瘤的30％～40％[1]。双表达淋巴瘤（DEL）是MYC和BCL2蛋白过表达但不伴相应基因重排的一类特殊亚群。根据世界卫生组织（WHO）分类标准，DEL的诊断需满足MYC蛋白表达≥40％且BCL2蛋白表达≥50％[1]–[2]。尽管DEL尚未被列为独立病理类型，但其在DLBCL中占比20％～30％，且多富集于活化的B细胞样亚型，与肿瘤微环境的免疫抑制、原发耐药等不良生物学行为密切相关[3]。

目前R-CHOP±维泊妥珠单抗方案仍是DLBCL的一线标准治疗方案。但大量研究证实，DE-DLBCL患者的预后显著劣于非DE-DLBCL患者[4]–[5]，其对传统免疫化疗方案的缓解率及长期生存率均不理想[6]。这种治疗挑战可能与DEL独特的分子机制相关，MYC/BCL2双表达与B细胞受体（BCR）信号通路的异常激活密切相关[7]，而布鲁顿酪氨酸激酶（BTK）正是该信号通路的核心调控节点[8]。BTK抑制剂（BTKi）通过不可逆共价结合BTK蛋白481位点半胱氨酸抑制其223位点酪氨酸磷酸化，进而抑制BTK活性。因此，BTKi有潜在的治疗DE-DLBCL的理论基础。

Phoenix研究首次探索BTKi联合R-CHOP方案治疗非生发中心（non-GCB）亚型DLBCL患者的疗效，其中伊布替尼联合R-CHOP的组合相较于安慰剂联合R-CHOP对照组在年龄小于60岁的MYC/BCL2双表达患者中显示出更优的无事件生存（EFS）和总生存（OS）[9]。然而，该研究整体人群未能达到主要终点，可能与老年患者治疗相关不良反应导致剂量调整有关[10]。这提示了对特殊患者人群筛选策略的精细化和新一代BTKi使用优化的需求。

因此，本研究着眼于新一代高选择性BTKi（包括泽布替尼、奥布替尼、阿可替尼），并通过回顾性分析35例初治DE-DLBCL患者接受BTKi联合R-CHOP方案的疗效与安全性数据，提供针对这一高危人群个体化治疗的医学证据。

## 病例与方法

1. 病例：本研究为回顾性队列研究，回顾性分析2021年6月至2024年6月于南京医科大学第一附属医院（江苏省人民医院）血液科就诊的接受BTKi+R-CHOP方案或R-CHOP方案治疗的初诊DE-DLBCL患者的结局。患者纳入标准：①根据WHO 2022版淋巴造血系统肿瘤分类标准诊断为DLBCL[1]；②免疫组织化学染色结果判断DEL：MYC蛋白表达≥40％，BCL2蛋白表达≥50％；③既往未接受过系统性抗淋巴瘤治疗；④完成至少4个周期治疗并具有可评估疗效。排除标准包括原发中枢神经系统DLBCL、原发性纵隔大B细胞淋巴瘤、EB病毒阳性DLBCL、双打击淋巴瘤或高级别B细胞淋巴瘤。研究共纳入95例DE-DLBCL患者，收集基线人口统计学数据和临床特征包括性别、年龄、美国东部肿瘤协作组（ECOG）评分、Ann Arbor分期、B症状（发热、盗汗、乏力或体重下降）、LDH、结外累及部位、国际预后指数（IPI）评分［年龄>60岁、Ann Arbor Ⅲ～Ⅳ期、ECOG评分≥2分、结外病灶>1个、LDH>正常值上限（ULN）］、免疫组织化学染色指标（MYC、BCL2、BCL6、Ki-67）。影像学资料包括治疗过程中的^18^F-氟代脱氧葡萄糖（FDG）PET-CT显像资料。本研究已获得南京医科大学第一附属医院伦理委员会的批准（批件号：2025-SR-551），纳入患者均签署知情同意书。

2. 治疗方案：R-CHOP方案，具体用药包括：利妥昔单抗375 mg·m^−2^·d^−1^静脉滴注，第0天；环磷酰胺750 mg·m^−2^·d^−1^静脉滴注，第1天；多柔比星50 mg·m^−2^·d^−1^（多柔比星脂质体30～40 mg·m^−2^·d^−1^）静脉滴注，第1天；长春新碱1.4 mg·m^−2^·d^−1^（最大剂量2 mg/次）静脉推注，第1天；泼尼松60 mg·m^−2^·d^−1^（分2次）口服第1～5天。治疗周期为21 d，重复6～8个周期。BTKi用法如下：泽布替尼160 mg早晚口服；或奥布替尼150 mg每日1次口服；或阿可替尼100 mg早晚口服。BTKi持续口服给药，每21天为1个疗程。参照NCCN指南，中枢神经系统（CNS）IPI评分4～6分或特殊部位（肾、肾上腺、睾丸、乳腺）受累患者均进行了4～6个周期CNS三联鞘内注射预防（甲氨蝶呤12.5 mg、阿糖胞苷35 mg、地塞米松5 mg）。

3. 疗效和安全性评估：疗效评价标准根据2014版Lugano疗效评定标准，分为完全缓解（CR）、部分缓解（PR）、疾病稳定（SD）和疾病进展（PD）。疗效评估时间点在治疗中期、诱导治疗结束时以及治疗结束后的2年内每3个月1次。治疗期间发生的所有不良事件（AE），均按照美国国家癌症研究所不良事件通用术语标准5.0版进行评估。

4. 随访和研究终点：通过住院病历、门诊就诊记录和电话对所有患者进行随访，随访截止日期为2025年6月30日，中位随访时间30（6～47）个月。主要研究终点为客观缓解率（ORR），为CR率和PR率之和。次要研究终点包括无进展生存（PFS）期和OS期。PFS期定义为从诊断至肿瘤进展/复发、任何原因死亡或末次随访的时间。OS期定义为从诊断至任何原因死亡的时间或末次随访的时间。

5. 统计学处理：采用Graphpad prism 10软件和R Studio 4.3.2版进行统计学分析。患者的临床病理特征、疗效和AE数据采用描述性统计方法进行总结。偏态分布的计量资料以*M*（范围）表示，分类变量以例数（百分比）表示。分类资料的组间比较采用卡方检验或Fisher精确概率法。生存分析采用Kaplan-Meier法，Log-rank检验进行组间比较。以*P*<0.05为差异具有统计学意义。

## 结果

1. 入组患者的临床特征：本研究共纳入95例DE-DLBCL患者，其中BTKi+R-CHOP组35例，R-CHOP组60例。两组患者的基线临床特征比较见表1。所有纳入比较的变量，包括性别、年龄、细胞起源、Ann Arbor分期、B症状、ECOG评分、IPI评分、结外受累部位、骨髓侵犯、LDH水平及Ki-67增殖指数，其组间差异均无统计学意义（*P*值均>0.05），表明两组基线均衡，具有可比性。BTKi+R-CHOP组，中位年龄61（29～73）岁，non-GCB亚型（97.1％）、晚期（Ⅲ～Ⅳ期，74.3％）和高肿瘤负荷（IPI评分3～5分者占51.4％）患者比例较高。

**表1:** 95例双表达弥漫大B细胞淋巴瘤患者的临床及生物学特征［例（％）］

临床特征	BTKi+R-CHOP组（35例）	R-CHOP组（60例）	*χ*^2^值	*P*值
性别			0.951	0.330
男	18（51.4）	37（61.7）		
女	17（48.6）	23（38.3）		
年龄			0.075	0.785
≥60岁	20（57.1）	36（60.0）		
<60岁	15（42.9）	24（40.0）		
细胞起源			2.879	0.090
GCB	1（2.9）	10（16.7）		
non-GCB	34（97.1）	50（83.3）		
Ann Arbor分期			1.990	0.158
Ⅰ～Ⅱ期	9（25.7）	24（40.0）		
Ⅲ～Ⅳ期	26（74.3）	36（60.0）		
B症状			0.148	0.701
有	16（45.7）	25（41.7）		
无	19（54.3）	35（58.3）		
ECOG评分			2.130	0.144
0～1分	24（68.6）	49（81.7）		
≥2分	11（31.4）	11（18.3）		
IPI评分			0.850	0.356
0～2分	17（48.6）	35（58.3）		
3～5分	18（51.4）	25（41.7）		
结外受累部位			0.424	0.809
0个	7（20.0）	10（16.6）		
1个	14（40.0）	22（36.7）		
≥2个	14（40.0）	28（46.7）		
骨髓侵犯			0.076	0.782
有	6（17.1）	9（15.0）		
无	29（82.9）	51（85.0）		
LDH水平			0.113	0.736
≤250 U/L	18（51.4）	33（55.0）		
>250 U/L	17（48.6）	27（45.0）		
Ki-67增殖指数			0.022	0.883
≥80％	25（71.4）	42（70.0）		
<80％	10（28.6）	18（30.0）		

**注** BTKi：布鲁顿酪氨酸激酶抑制剂；R-CHOP：利妥昔单抗+环磷酰胺+多柔比星/多柔比星脂质体+长春新碱+泼尼松；GCB：生发中心来源；non-GCB：非生发中心来源；ECOG：美国东部肿瘤协作组；IPI：国际预后指数；LDH：乳酸脱氢酶

2. 疗效与生存分析：95例患者均至少完成4个周期治疗，中位化疗周期数为8（4～8）个。BTKi+R-CHOP组中位化疗周期数为8（4～8）个，R-CHOP组中位化疗周期数为8（4～8）个，组间差异无统计学意义（*P*＝0.084）。BTKi+R-CHOP组中，25例（71.4％）接受泽布替尼联合R-CHOP（zR-CHOP）方案治疗，6例（17.1％）接受奥布替尼联合R-CHOP（oR-CHOP）方案治疗，4例（11.4％）接受阿可替尼联合R-CHOP（aR-CHOP）方案治疗。中期评估显示，BTKi+R-CHOP组的ORR为97.1％（34/35），21例（60.0％）患者获得CR、13例（37.1％）患者获得PR、1例（2.9％）患者获得PD。诱导治疗结束后，该组ORR为94.3％（33/35），32例（91.4％）患者获得CR、1例（2.9％）患者PD。而R-CHOP组诱导治疗结束后的ORR为71.7％（43/60），CR率为65.0％（39/60）。BTKi+R-CHOP组在ORR（*P*＝0.008）和CR率（*P*＝0.006）上均优于R-CHOP组。诱导治疗结束后，BTKi+R-CHOP组中有4例（11.4％）患者接受了自体造血干细胞移植（auto-HSCT）巩固治疗；3例（8.6％）患者因自身原因未进行后续维持治疗，30例（85.7％）患者均接受BTKi口服维持治疗。BTKi治疗的中位时间为26（4～32）个月。

如表2所示，诱导治疗结束时，各临床亚组中BTKi+R-CHOP方案均呈现较高缓解率，组间比较差异均无统计学意义（*P*值均>0.05）。≥60岁患者（20例）CR率与<60岁患者（15例）相当（90.0％对93.3％，*P*>0.99）；IPI评分为3～5分的患者（18例）CR率低于0～2分的患者（17例）（83.3％对100％，*P*＝0.229）；Ann Arbor Ⅲ～Ⅳ期的患者（26例）CR率低于Ⅰ～Ⅱ期的患者（9例）（88.5％对100％，*P*＝0.553）；值得注意的是，多结外受累（≥2个部位）患者（14例）CR率低于无/单结外受累患者（21例）（78.6％对100％，*P*＝0.055），虽差异无统计学意义但提示临床趋势。尽管样本量存在差异，各BTKi亚组均显示良好疗效。zR-CHOP组（25例）CR率为88.0％，oR-CHOP组（6例）CR率为100％，aR-CHOP组（4例）CR率为100％，组间差异无统计学意义（*P*>0.99）。所有亚组ORR均>85％，提示BTKi+R-CHOP方案对不同特征患者均具有良好疗效。

**表2:** 诱导治疗结束后35例双表达弥漫大B细胞淋巴瘤患者的亚组疗效分析［例（％）］

临床特征及治疗选择	例数	CR率	*P*值	ORR	*P*值
年龄			>0.99		>0.99
≥60岁	20	18（90.0）		18（90.0）	
<60岁	15	14（93.3）		14（93.3）	
IPI评分			0.229		0.523
0～2分	17	17（100）		17（100）	
3～5分	18	15（83.3）		16（88.9）	
Ann Arbor分期			0.553		>0.99
Ⅰ～Ⅱ期	9	9（100）		9（100）	
Ⅲ～Ⅳ期	26	23（88.5）		24（92.3）	
B症状			0.086		0.201
无	19	19（100）		19（100）	
有	16	13（81.3）		14（87.5）	
结外受累部位			0.055		0.153
0～1个	21	21（100）		21（100）	
≥2个	14	11（78.6）		12（85.7）	
Ki-67增殖指数			0.541		>0.99
≥80％	25	22（88.0）		23（92.0）	
<80％	10	10（100）		10（100）	
治疗方案			>0.99		>0.99
zR-CHOP	25	22（88.0）		23（92.0）	
oR-CHOP	6	6（100）		6（100）	
aR-CHOP	4	4（100）		4（100）	

**注** CR：完全缓解；ORR：客观缓解率；IPI：国际预后指数；z：泽布替尼；R-CHOP：利妥昔单抗+环磷酰胺+多柔比星/多柔比星脂质体+长春新碱+泼尼松；o：奥布替尼；a：阿可替尼；所有分类变量的组间比较均采用Fisher精确检验，故无*χ*^2^值

随访截至2025年6月30日，中位随访时间为30（6～47）个月，BTKi+R-CHOP组共3例患者死亡：1例中期PD患者于PD后3个月死亡；1例患者在治疗期间因新型冠状病毒感染死亡，最佳疗效评价为PR；1例CR患者于随访期复发，经CAR-T细胞治疗后合并肺部感染死亡，判断与泽布替尼的使用无关。该组患者中位PFS期和OS期均未达到。1年和2年PFS率分别为88.5％（95％ *CI*：78.5％～99.8％）和85.1％（95％ *CI*：73.8％～98.1％）。1年及2年OS率均为94.3％（95％ *CI*：86.9％～100％）。R-CHOP组1年、2年PFS率分别为61.7％（95％ *CI*：50.5％～75.3％）和48.8％（95％ *CI*：37.4％～63.6％）；1年、2年OS率分别为80.0％（95％ *CI*：70.5％～90.8％）和65.1％（95％ *CI*：53.7％～78.8％）。BTKi+R-CHOP组在PFS（*P*＝0.002）和OS（*P*＝0.010）上均优于R-CHOP组。单因素分析结果显示，年龄≥60岁、IPI评分3～5分、B症状、LDH升高、β_2_微球蛋白、BCL6表达与PFS均无关联（*P*值均>0.05）。

3. 安全性分析：BTKi联合R-CHOP方案治疗总体耐受性良好。常见的≥3级血液学AE：中性粒细胞减少症（10例，28.6％）、血小板减少（6例，17.1％）、贫血（4例，11.4％）、发热性中性粒细胞减少症（3例，8.6％）。最常见的非血液学AE是≥3级肺部感染（5例，14.3％）。其余1～2级非血液学AE：黏膜出血（3例，8.5％）、恶心呕吐（2例，5.7％）、皮疹（2例，5.7％）和关节痛（1例，2.9％）。随访过程中观察到有3例患者合并基础心脏病（冠状动脉粥样硬化性心脏病1例、心房颤动2例），但未观察到≥3级治疗相关心血管事件。值得注意的是，泽布替尼相关黏膜出血均为1～2级（3/35，8.5％），无严重或致死性出血事件发生。

## 讨论

本研究基于真实世界数据，对BTKi联合R-CHOP方案治疗初治DE-DLBCL的疗效与安全性进行评估。结果显示该联合方案在诱导治疗结束时ORR为94.3％，CR率为91.4％，均高于本中心同期接受单纯R-CHOP方案治疗的患者。该亚组均维持高缓解率，并显著提升短期缓解指标，1年和2年PFS率分别达88.5％和85.1％，1年及2年OS率均为94.3％，安全性可控，为高危亚型患者提供了新的治疗选择。

MYC和BCL2的共表达现象不仅增强肿瘤细胞增殖和抗凋亡能力，还通过持续激活BCR信号通路，导致肿瘤细胞对R-CHOP方案的敏感性降低[11]。既往Phoenix研究数据支持这一观点，标准R-CHOP方案治疗DE-DLBCL的CR率仅为64.9％，即使联合伊布替尼也仅提升至67.5％[12]。中期治疗反应对患者长期预后具有重要预测价值。一项纳入46例DLBCL患者的回顾性研究（中位随访65.9个月）显示，代谢部分缓解（mPR）组患者的OS率、无复发生存（RFS）率和免于疾病进展期（FFDP）均显著低于代谢完全缓解（CMR）组（*P*<0.05）[13]。另一项研究进一步证实，中期PET-CT阳性患者的2年PFS率为50.0％，显著低于PET-CT阴性患者（86.4％，*P*＝0.001）[14]。以上结果提示，中期评估为PR可能预示着疾病快速进展风险增加和远期预后不良，因此应考虑强化后续治疗方案。近期一项回顾性研究比较了zR-CHOP方案和R-CHOP方案在DE-DLBCL中的疗效，结果显示zR-CHOP组在4个周期（82.6％对50.9％，*P*＝0.019）和6个周期（95％对65.2％，*P*＝0.025）后的CR率均高于R-CHOP组[15]。虽然两组ORR差异均无统计学意义，但CR率的显著提升提示BTKi可能通过诱导深度缓解改善预后。本研究结果显示，患者诱导治疗结束时ORR为94.3％，CR率为91.4％，高于本中心同期接受R-CHOP治疗的对照组。短期疗效指标尤其是CR率较历史数据亦显著改善。相较于第一代BTKi伊布替尼，新一代药物具有更高的靶点选择性和更佳的药代动力学特征，可能带来更少的脱靶效应、更高的安全性以及潜在的疗效优势[16]。BTKi的及时加入可能克服DE-DLBCL的化疗耐药性，提高肿瘤深度缓解以及长期的生存获益。

亚组分析显示BTKi联合方案在年龄、IPI评分、Ann Arbor分期及结外受累程度的亚组患者中ORR均超过85％。结外受累的解剖学分布和数量对疾病预后具有重要影响。中国一项大型多中心回顾性研究（1 085例）数据表明，结外受累（EN-DLBCL）在DLBCL患者中的发生率为37.4％，且此类患者的预后显著差于结内受累（N-DLBCL），其5年OS率和PFS率分别为56.9％和49.0％[17]。近期临床研究显示，新型BTKi联合方案可能改善这类高危患者的预后。zR-CHOP方案治疗伴结外受累的初诊non-GCB亚型DLBCL患者可获得91.3％的ORR和82.61％的CR率，2年累积PFS率和OS率分别为74.0％和88.5％[18]。oR-CHOP方案在高危伴结外累及的non-GCB亚型DLBCL中也显示出良好的抗肿瘤活性（ORR为88.6％、CR率68.6％），2年PFS率和OS率分别为68.6％和87.5％[19]。本研究97.1％（34/35）的患者为non-GCB亚型，40％（14/35）伴多个结外受累（≥2个部位），多结外受累组患者诱导治疗结束时的ORR为85.7％，CR率为78.6％。虽然差异无统计学意义，但多结外受累组的CR率较无/单结外受累组（100％）低，可能与多结外受累患者的肿瘤生物学特性相关。BTKi对这类亚群可能具有选择性优势，但仍需延长随访时间以明确CR率提升是否转化为生存获益。值得注意的是，基于PET-CT评估的结外受累部位数量已被证实是DLBCL患者CNS复发的预测因素[20]。此外，双表达状态也与CNS复发密切相关。有数据显示，与非DE-DLBCL相比，DE-DLBCL在初诊2年后CNS复发风险显著增高（9.7％对2.2％，*P*<0.001）[21]。本研究zR-CHOP组随访期间观察到1例ⅠE期原发乳腺DLBCL合并MYC基因重排患者在治疗2年后出现孤立性CNS复发。尽管BTKi类药物具有一定程度的血脑屏障穿透能力，但对于存在高危因素（如MYC重排、特定结外部位受累）的患者，其预防CNS复发的效果可能有限。对于具有CNS复发高危因素的患者，需要考虑更加优化的预防策略。

本研究中长期生存分析显示，中位随访30个月，BTKi+R-CHOP组中位PFS期和OS期均未达到，1年及2年PFS率分别为88.5％和85.1％，不仅显著优于既往文献中R-CHOP方案作为标准治疗所报告的2年EFS率（75％）[22]，也明显高于本中心同期采用单纯R-CHOP方案治疗DE-DLBCL患者的2年PFS率（48.8％）。尽管样本量有限，这一结果与近期多项研究报道的BTKi联合化疗生存改善趋势吻合[23]–[24]。单因素分析未发现年龄、IPI评分、LDH升高等传统高危因素与PFS的显著关联，可能与随访时间较短或样本量不足有关，需延长随访时间进一步验证。本研究中BTKi+R-CHOP组3例死亡患者年龄皆≥70岁，这提示我们对高龄患者需个体化调整治疗强度并加强支持治疗。

血液学不良反应是BTKi联合化疗最常见的AE，本研究中3～4级中性粒细胞减少症发生率较低。可能与使用新一代BTKi、骨髓受累率低（17.1％，6/35）及长效粒细胞集落刺激因子（G-CSF）预防性应用相关。肺部感染（14.3％）是最常见的非血液学AE，且本队列未观察到BTKi相关≥3级心血管事件或严重出血事件发生。

本研究存在一定局限性，因单中心设计、样本量较小及中位随访时间较短，可能影响生存分析。此外，不同BTKi的亚组样本量不均衡，尽管均显示高缓解率，但差异仍需更大样本验证。同时，本研究缺乏二代测序基因突变分析结果，导致无法基于基因突变特征精准筛选BTKi治疗的潜在获益人群。未来可开展多中心Ⅲ期随机对照试验，延长随访时间，明确长期生存获益；整合基因突变谱及动态疗效监测指标，建立个体化治疗预测模型。

综上所述，BTKi+R-CHOP方案在DE-DLBCL患者中展现显著抗肿瘤活性与可控安全性，有望成为这一高危亚型的一线治疗新选择，为后续大规模临床试验提供依据。同时支持进一步探索靶向药物联合化疗在DLBCL精准治疗时代的应用价值。
